# Interest of Tumor-Specific CD4 T Helper 1 Cells for Therapeutic Anticancer Vaccine

**DOI:** 10.3390/vaccines3030490

**Published:** 2015-06-30

**Authors:** Jeanne Galaine, Christophe Borg, Yann Godet, Olivier Adotévi

**Affiliations:** 1 INSERM UMR1098, Besançon cedex F25020, France; E-Mails: jeanne.galaine@edu.univ-fcomte.fr (J.G.); christophe.borg@efs.sante.fr (C.B.); yann.godet@univ-fcomte.fr (Y.G.); 2 Université de Franche-Comté, Besançon cedex F25020, France; 3 EFS Bourgogne Franche-Comté, Besançon cedex F25020, France; 4 Department of Medical Oncology, University Hospital of Besançon, Besançon cedex F25020, France

**Keywords:** CD4 T cell, tumor antigen, helper peptide, cancer vaccine

## Abstract

Nowadays, immunotherapy represents one promising approach for cancer treatment. Recently, spectacular results of cancer immunotherapy clinical trials have confirmed the crucial role of immune system in cancer regression. Therapeutic cancer vaccine represents one widely used immunotherapy strategy to stimulate tumor specific T cell responses but clinical impact remains disappointing in targeting CD8 T cells. Although CD8 T cells have been initially considered to be the main protagonists, it is now clear that CD4 T cells also play a critical role in antitumor response. In this article, we discuss the role of tumor antigen-specific CD4 T cell responses and how we can target these cells to improve cancer vaccines.

## 1. Introduction

The proof of concept of cancer immunotherapy efficacy has reached an important inflection point in the history of cancer therapy. Encouraging responses are consistently being reported for a broad range of human cancers with immunotherapies agents, notably so-called immune checkpoint inhibitors (CTLA-4, PD-1 and PDL-1) [1]. The impressive response obtained with immune checkpoint inhibitors indicated that the presence of preexisting antitumor immune response is required for their efficacy. These emerging clinical data highlight the critical role of adaptive T cell immunity and suggest that cancer immunotherapy is likely to become a key part of the clinical management of cancer [2,3].

Therapeutic cancer vaccine is an active immunotherapy whose primary aim is to induce or enhance an adaptive antitumor immunity, notably T cells against tumor cells. Many vaccine approaches such as peptides, proteins, viruses, DNA, dendritic cells, and tumor cell lysates have been used to sustain T cell responses *in vivo* [4]. Although therapeutic cancer vaccines have very good safety and tolerability in clinical settings, their efficacy was limited on several accounts, notably by immune suppression microenvironment within the tumor [5]. The challenge now is to develop new and optimized therapeutic vaccines for harnessing tumor specific T cell responses*.* Although cytotoxic CD8 T cells have been considered to be the main protagonists in the production of anti-tumor therapeutic effects resulting from vaccines, increasingly, several aspects of CD4 T-cell biology suggest that this T-cell population can effectively be used for cancer immunotherapy. Hence, previous studies demonstrated that CD4 T cells could be involved in the efficacy of many cancer immunotherapies [6,7,8,9,10]. Several approaches have been developed to stimulate antitumor CD4 T cell immunity, during therapeutic cancer vaccines. The most common strategy relies on the use of a helper peptide derived either from non-tumor antigens or tumor antigens, and more recently from neoantigens [11].

## 2. Critical Roles of CD4 Helper T Lymphocytes in Antitumor Immunity

CD4 T cells orchestrate a broad range of immune responses and are equipped to differentiate into multiple sublineages, which can induce and maintain immune responses against tumor antigens. Although originally defined as Th1 and Th2 subsets, new Th CD4 T cells subsets emerged the last decades such as suppressive regulatory T cells (Tregs) and pro-inflammatory Th17, and more recently for Th9, Th22 and follicular helper T cells [12,13,14,15,16,17]. Although Th1 and Th2 subsets are initially considered as the most stable and mutually exclusive lineages, it now appears that they depend on the differentiation state [18]. Likewise, Th17 and Treg subsets do not represent stable differentiation processes and retain plasticity allowing them to adapt to different environments [18,19].

Among these various Th subpopulations, the Th1 subset that produces interferon-gamma (IFN-γ), tumor necrosis factor-alpha (TNF-α) and interleukine-2 (IL-2), plays a clear antitumor role by orchestrating cell-mediated immunity against cancer cells [20,21]. Earlier studies in mouse model showed that successful generation of antitumor CD8 T cell responses depend on the presence of CD4 T cells [20,22]. The induction of DC activation represents one major helper mechanism used by Th1 cells to sustain antigen presentation and to provide costimulatory signals such as CD40-CD40L to effector CD8 T cells [23,24,25]. Importantly, it has also been shown that Th1 cells enhance the CD8 T cells infiltration into the tumor [26,27,28,29]. This effect was mediated by IFN-γ dependent production of chemokines such as CXCL9 and CXCL10 [27,30]. However, CD4 Th1 cells also exhibit CD8 T cells’ independent antitumor activity. The IFN-γ secreted by Th1 cells exerts anti-proliferative, pro-apoptotic actions and inhibit angiogenesis in tumor cells [31]. Furthermore, Th1 cells also recruit and activate inflammatory cells (macrophages, granulocytes, eosinophils and NK cells) in around the tumor [20,21]. Furthermore, IFN-γ induces the upregulation of major histocompatibility complex (MHC) molecules on tumor cells leading to enhanced effector T cells recognition [31]. This mechanism enables MHC Class II restricted killing independently of B, NK or other T cells [32,33]. Indeed, some CD4 Th1 cells have also direct tumor-recognizing ability [34]. They are able to kill MHC-II^+^ tumors through perforine and granzyme, TNF-related apoptosis inducing ligand (TRAIL) receptor and Fas/Fas ligand (FasL) pathways [20,21]. Finally, CD4 T cells can also provide help to themselves. Th-Th interaction enables the activation of CD4 T cells specific for a poorly immunogenic epitope [11].

In cancer patients, spontaneous CD4 T cell responses against tumor antigens have been detected in several studies [28,35,36,37,38,39,40,41,42,43]. Accordingly, a high density of tumor-infiltrating Th1 cells has been identified as a good prognostic marker in several human cancers [44,45]. On the other hand, subsets such as Th2, Tregs, or, under some circumstances, Th17 cells, may have tumor-promoting activity, which may need to be curtailed to obtain optimal antitumor responses [46]. Finally, only Th1 immune response has been shown to mediate bona fide anticancer effects in cancer patients, providing a strong rationale to develop antitumor Th1 immunity-stimulating immunotherapy, which is supported by many clinical trials.

## 3. Interest to Stimulate CD4 T Helper 1 Response for Therapeutic Cancer Vaccine

The role of CD4 Th cells in cancer vaccine is supported by early studies in animals showing that the depletion of CD4 T cells inhibited vaccine-induced protective immunity [47,48,49]. Based on the critical role of CD4 Th1 cells in antitumor immunity, we believe that effectiveness of cancer vaccine could be greatly enhanced by stimulating suitable tumor-reactive Th1 immunity, making a “good” tumor microenvironment for the immune effector cells action (CD8 T cells, NK cells and M1 macrophages) and a “bad” one for the immune suppressive cells (Tregs, MDSC, *etc.*). Hence, a reasonable alternative to boost helper response is to focus on MHC class II-binding peptides from tumor antigens.

To do this, we propose that four major criteria have to be considered to select tumor-specific Th1-inducer helper peptides for cancer vaccine: (i) the helper peptide must derive from a shared overexpressed tumor antigen; (ii) the tumor antigen should have crucial role in the oncogenesis to avoid immune escape; (iii) the tumor-reactive helper peptide should be highly promiscuous to immunize a large segment of the human population; and (iv) the helper peptides should preferentially stimulate Th1 immunity to prevent the induction of detrimental Th responses [50]. Several antigens with some of these properties have already been described such as human telomerase reverse transcriptase (hTERT) [51], HER2 [52], survivin [53], NY-ESO-1 [54], and MUC1 [55]. In order to stimulate CD4 Th1 responses in cancer patients, increasing attention has been focused on identifying MHC class II-restricted epitopes from relevant human tumor antigens to actively target these cells for cancer vaccine [56,57]. However, only a few tumor-reactive helper peptides are evaluated in clinical settings.

One common approach to stimulate CD4 T cell helper response is the use of xenogenic or non-tumor antigens that recall immune memory or provide a non-specific help. The synthetic helper peptide PADRE derived from keyhole limpet hemocyanin (KLH) and the tetanus toxoid-derived helper peptide are commonly used in anticancer vaccines [58,59]. In recent randomized vaccine trials, melanoma patients were vaccinated with multi melanoma-derived cytotoxic T lymphocytes (CTL) peptides vaccine either in the presence of a tetanus-derived helper peptide or with melanoma-derived helper peptides [8,60]. In contrast to the group that received CTL peptides and tetanus helper peptide, a high clinical objective response rate (ORR) was observed in patients treated with Melanoma-derived helper peptides [8,9]. Interestingly, significant ORR was also observed when melanoma-helper peptides were used alone as compared to CTL-peptides vaccination. One possible explanation for these observations is that helper peptides unrelated to tumor antigens may be ineffective in guiding effector CD8 T cells within the tumor [61].

These observations are in line with previous reports by using tumor-derived helper peptides such as HER2-neu and hTERT [62,63]. Hence, a hTERT-derived helper peptide vaccine called GV1001 has been evaluated in many cancers [64]. In lung cancer patients GV100 vaccination induced a durable T-cell memory response and increased survival in immune responders [63]. However GV1001 vaccination fails to reach the main end point overall survival in pancreatic adenocarcinoma [65]. Recently, our group described novel antitumor Th1-inducer peptides derived from hTERT also called Universal Cancer Peptides (UCP) [28,37]. We found that the immunoprevalence and magnitude of UCP-specific Th1 responses are higher than GV1001 in several cancers (manuscript submitted). Thus, we believe that UCPs are attractive helper peptides for therapeutic cancer vaccine and a clinical trial with these peptides is planned to be conducted in lung cancer.

Instead of vaccinating with a mix of Th and CTL epitopes, it was proposed later, to conjugate the two epitopes to form a single longer linear hybrid peptide named synthetic long peptide (SLP) [66,67,68]. This strategy allowed the improvement of the immunogenicity by increasing the duration of *in vivo* epitope presentation [69] and by inducing a broader immune response with the stimulation of both CD8 and CD4 T cells [49,70,71]. The efficacy of SLP-based vaccination has been shown in pre-clinical models [72,73] and in patients with cervical cancer [74,75].

Recently, Aarntzen *et al.* reported that the use of tumor-derived helper peptides greatly improved the efficacy responses of DC-based vaccination [76]. Furthermore, there exists clear evidence for epitope spreading, following immunization of cancer patients with tumor-reactive helper peptides [77,78,79]. Collectively, these results highlighted the necessity of tumor-specific CD4 T cell stimulation for vaccine success [49,80,81,82].

## 4. Emerging Personalized Vaccine Using CD4 Helper Peptides from Neoantigens

Conventional cancer vaccines have targeted shared self-antigens with the advantage of being universally available for patients. However central and peripheral tolerance could deplete the most reactive specific T cells. In this regard, it seems interesting to design cancer vaccines targeting non-self-tumor-specific antigens like those emerging from mutations, namely cancer neoantigens.

Despite the impressive work accomplished to monitor T-cell reactivity against large collections of shared epitopes, the cumulative fraction of tumor-infiltrating T cells that were reactive with these epitopes showed to be low [83,84]. One possible explanation for this observation could be that neoantigens specific T cells form a significant component of tumor specific T cell responses and that these studies were focused on CD8 T cells. Prior works have clearly established that neoantigens can be recognized in human cancers, and that reactivity against patient-specific antigens can be stronger than the one against shared antigens [7,85]. Neoantigens can arise from mutation or cancer specific splice isoforms. Recurrent alternative splicing events have been identified [86] and some of them are immunogenic [38,87]. However, until now, the major known somatic alterations in the cancer genome leading to neoantigens include nucleotide substitution mutations, small insertion/deletions (indels) and chromosomal rearrangements. Missense mutations are the most frequent DNA modifications observed [88] and most recent works focused on these mutations within exonic region [10,89,90]. However, insertions, deletions and chromosomic translocations are believed to be more immunogenic than missense as they could induce frameshifts and the immunogenicity of splice site mutations is poorly studied. Despite these limits, several epitopes derived from neoantigens are described [10,89,90,91] and neoepitopes derived from these mutations preferentially stimulate CD4 T cell responses [10]. In addition, the efficacy of checkpoint inhibitors seems to rely on mutation load [92,93]. These recent observations pointed out once more, the role of CD4 T cells in antitumoral immune response and the interest in targeting them to increase clinical benefit of cancer vaccines.

Neoantigens appear advantageous for antitumor vaccine because CD4 T cell precursors exist in a nontolerized form within the repertoire of the nonimmunized individual. With exon and RNA sequencing, algorithms already exist to rapidly match neoepitopes with MHC alleles, thereby facilitating the identification of peptides for personalized cancer medicine [94].

However, neoepitopes targeting strategies meet several hurdles. Firstly, data from genome sequencing and single cell sequencing analysis have revealed a surprising tumor genetic heterogeneity [95,96]. Thus, targeting a unique neoantigen would probably lead to selection of antigen non-expressing tumor cells. Secondly, assessing the immunogenicity of each neoepitopes is not reasonably applicable on a large scale. To bypass these hurdles, Kreiter *et al.* have proposed to select strong HLA binders that are highly expressed [10]. However, the number of neopitopes that are needed is not determined in humans. Thirdly, vaccination with neoepitopes could lead to cross-reactions, due to peptide mimicry, inducing autoimmunity as it has been observed for MAGE-3 [97]. Nonetheless, clinical trials targeting neoepitopes are ongoing (NCT00683670, NCT02287428, NCT01970358, NCT02129075, NCT02035956) and results will help us to establish the relevance of this vaccine strategy.

## 5. Conclusions and Perspectives

Owing to the central role of CD4 T cells in antitumor immunity, targeting MHC-II restricted antigens will doubtless improve cancer vaccine efficacy in the future. Beyond the effector immune cells, the choice of antigens remains crucial, and each of them presents advantages as well as drawbacks. Shared antigens are broadly relevant in a non-personalized vaccination strategy while neoantigens are patient and tumor-specific. However, neoantigens-specific T cells are not selected in the thymus.

The success story of checkpoint inhibitors, anti-cytotoxic T lymphocytes antigen 4 (CTLA-4) (ipilimumab) and anti-programmed death 1 PD-1 (nivolumab, pembrolizumab), reinforces the notion that it exists a preexisting antitumor immune response but it fails to eradicate all tumor cells because of immune suppressive establishment in tumor microenvironment [1,3,98]. Thus, immunotherapy strategy, particularly vaccine strategy, could be more effective when combined with molecules, which break cancer immunosuppression such as chemotherapy depleting Tregs and myeloid-derived suppressor cells (MDSC) [99,100], tyrosine kinase inhibitor [101] or antibody directed against checkpoint receptors [102,103].
